# Addenbrooke's Hospital

**Published:** 1903-02-14

**Authors:** 


### ADDENBROOKE'S HOSPITAL.

### THE NEW SCHEME OF MANAGEMENT.

THE following circular has been issued to the Governors of Addenbrooke's Hospital by the advisory committee:—You are probably aware that application has been made by the General Court of Governors to the Charity Commissioners to obtain from Parliament a new scheme for the government of the Hospital.

The Act of 1766 gives the management of the Hospital to the Governors at large, who now number about 600. It is felt that the Institution has outgrown the limits within which this mode of government is efficient, and a Special General Court of Governors has sanctioned an attempt to improve upon it by introducing the principle of a responsible Executive, elected by the Governors, and charged with the administration. This change, it was found, could not be made without fresh legislation, which would cause great expense to the Hospital, if promoted directly by the Governors. If, however, it were promoted by the Charity Commissioners under the powers of "The Charitable Trusts Act, 1853," a new scheme might be obtained without expense; and this course has accordingly been adopted.

Under the scheme laid before the Charity Commissioners it is proposed that the administration of the hospital shall be conducted and carried on and all its affairs shall be managed by a general committee of 24 elected Governors, eight from residents in the borough, eight from residents in the county, and eight from resident members of the University, who are on the electoral roll, so as to make the committee as far as practicable representative of the borough, county, and University Governors respectively. Certain defined acts and proceedings of the committee are to be subject to confirmation by a General Court of the Governors; and the general committee is to bring up a report of its proceedings at each of the four quarterly courts. The members of the committee to hold office for two years, one half to retire annually.

In a letter dated June 21st, 1902, sent to Mr. Bonnett by Mr. Durnford, secretary of the Charity Commissioners, Mr. Durnford stated with reference to the scheme that the general scope and purpose of the proposals therein contained had the full support of the Charity Commissioners.

A protest against the scheme, signed by 14 Governors, has been sent in to the Commissioners, who intend to refer the matter of the scheme to one of their officers for local inquiry.

Management by a responsible executive, such as is proposed for Addenbrooke's, has become almost universal in the hospitals of this country; and we believe that it will conduce to the efficiency and economical administration of our hospital.

As it is most important that the Commissioners should be made acquainted with the views of the general body of Governors, we shall feel greatly obliged if you will kindly state on the enclosed postcard whether you are for, or against, the proposal to establish a responsible executive committee on the lines above suggested.

At the special General Court called to consider the matters referred to in this circular and held on the 26th ult., it was stated by the chairman, Mr. George Kett, that the poll had resulted in 192 votes being given in favour of the scheme and only 21 against it. In the result, after taking up a great deal of time by making speeches of inordinate length, the few and ancient Governors who are opposed to reform withdrew their opposition, and so there is at last good grounds for hope that Addenbrooke's Hospital may soon be granted a system of management on modern lines. Then, with the disappearance of the old bad system and its supporters, under which the finances of the hospital have gone from bad to worse, it is reasonable to hope that public confidence will be restored and adequate funds will then be provided.

